# Assessment of Marine Gill Disease in Farmed Atlantic Salmon (*Salmo salar*) in Chile Using a Novel Total Gross Gill Scoring System: A Case Study

**DOI:** 10.3390/microorganisms9122605

**Published:** 2021-12-16

**Authors:** Sophie Fridman, Smaragda Tsairidou, Nilantha Jayasuriya, Halina Sobolewska, Alastair Hamilton, Carlos Lobos, Ross D. Houston, Hamish Rodger, James Bron, Tharangani Herath

**Affiliations:** 1 Institute of Aquaculture, University of Stirling, Stirling FK9 4LA, UK; j.e.bron@stir.ac.uk; 2 The Roslin Institute and Royal (Dick) School of Veterinary Studies, University of Edinburgh, Edinburgh EH25 9RG, UK; smaragda.tsairidou@roslin.ed.ac.uk (S.T.); ross.houston@roslin.ed.ac.uk (R.D.H.); 3 Department of Animal Health, Behaviour and Welfare, Harper Adams University, Newport TF10 8NB, Shropshire, UK; njayasuriya@harper-adams.ac.uk (N.J.); therath@harper-adams.ac.uk (T.H.); 4 Noahgene Ltd., The e Centre, Cooperage Way, Alloa FK10 3LP, UK; halina@noahgene.com; 5 Hendrix Genetics, Villa ’de Körver’, Boxmeer, 695831 CK Spoorstraat, The Netherlands; alastair.hamilton@hendrix-genetics.com (A.H.); carlos.lobos@zoetis.com (C.L.); 6 PHARMAQ Analytiq Spa, Bernardino, Puerto Montt 1978, Chile; 7 VAI Consulting, Kinvara, H91 N9CT Galway, Ireland; hamish.dmr@gmail.com

**Keywords:** multifactorial gill disease, diagnostics, *Salmo salar*, gill health

## Abstract

Gill disorders have become more prevalent and widespread in finfish aquaculture in recent years. Their aetiology is often considered to be multifactorial. Effective diagnosis, control and prevention are hindered by the lack of standardised methodologies to characterise the aetiological agents, which produce an array of clinical and pathological presentations. The aim of this study was to define a novel gross pathological scoring system suitable for field-based macroscopic assessment of complex or multifactorial gill disease in farmed Atlantic salmon, using samples derived from a gill disease outbreak in Chile. Clinical assessment of gross gill morphology was performed, and gill samples were collected for qPCR and histology. A novel total gill scoring system was developed, which assesses gross pathological changes combining both the presumptive or healed amoebic gill disease (AGD) and the presence of other types of gill lesions. This scoring system offers a standardised approach to characterise the severe proliferative pathologies in affected gills. This total gill scoring system can substantially contribute to the development of robust mitigation strategies and could be used as an indicator trait for incorporating resistance to multifactorial gill disease into breeding goals.

## 1. Introduction

Gill disorders are a significant global challenge for the Atlantic salmon (*Salmo salar* L.) industry during the marine phase of farming [1]. Contributing factors include an array of aetiological agents that involve parasites, viruses, bacteria and non-infectious organisms (e.g., harmful phytoplankton species), as well as environmental and management practices, such as treatments, pumping, and in situ net cleaning. Improved understanding of the disease aetiology is key to treatment, control and prevention. However, this wide range of pathogens that may contribute to a complex array of clinical presentations, are difficult to differentially diagnose [2].

Gross pathological assessment using gill-scoring criteria provides a non-invasive and economically realistic method for assessing gill disease at a commercial level [3,4,5,6]. To date, the most commonly accepted scoring system for the proactive management of gill diseases which is used across some sections of the industry is a gross gill score system, which classifies gross clinical signs of amoebic gill disease (AGD), i.e., raised white mucoid spots and streaks/patches on the gill surface, scored on a scale of 0 to 5, with 0 being clear and 5 showing extensive coverage [6], and amended or alternate scales are utilized in different regions and companies for AGD (for example, a double gill scoring system was proposed by Persson, et al. [7]). However, the interpretation of the score with respect to the number of arches scored and the choice of arches (for example, scoring only the most severely affected arch [8]), has differed between operators. Hence, the validity of the interpretation of this subjective scoring system is under ongoing discussion, and the risks of possible underestimation of severity of disease have been highlighted [9,10]; these systems inevitably lower the weighting of early onset AGD where only one or two spots may be seen across the entire gill area.

Following these concerns, a more comprehensive approach to AGD monitoring was developed, clarifying the scoring system by suggesting the inclusion of all 16 gill arch faces [11]. The complexity of clinical presentations of gross gill pathology in cases of multifactorial gill diseases has resulted in a range of scoring systems, with presentations being described in a number of ways, e.g., proliferative gill disease (PGD) and complex gill disease (CGD) [2]. A standardised diagnostic approach to assess gross pathological changes in gills in farmed salmonids would assist surveillance and the design of control strategies. Such a practical tool for assessing and recording gill health in the field would also generate easily-measurable phenotypes for resistance to multifactorial gill disease, which can be used for improving disease resistance via selective breeding [12]. Therefore, the aim of this study was to define a novel total gross pathological gill scoring system, suitable for field-based macroscopic assessment of complex or multifactorial gill disease in farmed Atlantic salmon.

## 2. Materials and Methods

**Figure 1:**
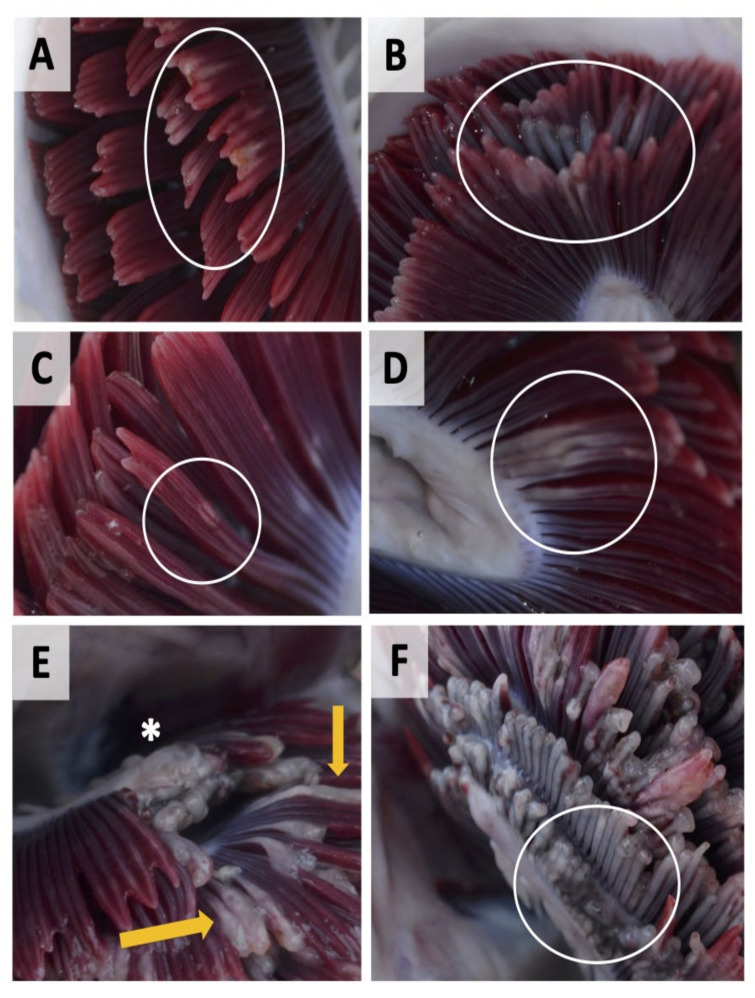
Examples of gross gill pathologies in farmed Atlantic salmon in Cupquelan Fjord displaying multifactorial gill disease. (**A**) superficial shortening of gill filaments with varying degrees of gill pallor and mucus accumulation (white circle); (**B**) more extensive erosion of filaments (white circle); (**C**) presence of discrete focal spots or streaks on the gill filaments (white circle); (**D**) multi-filamental patch with mucus accumulation (white circle); (**E**) grossly thickened/swollen gill filaments (yellow arrows), area of total filament erosion and necrotic patches (asterisk); (**F**) extensive filament erosion, pallor and areas of melanisation (white circle).

In early 2019, an outbreak of gill disease was reported in the Cupquelan Fjord system in the XIth Region in southern Chile. The affected fish population were at harvest size, and had not received any relevant treatment. Clinical assessment of gross morphology of fish gills was carried out between 15–17 April 2019. Gill samples were collected for PCR-based aetiology assessment and histomorphological assessment from 42 lethally sampled fish of average weight 7686 ± 1402 g. Prior to sampling, fish were fasted for at least two days and anaesthetised using benzocaine as part of commercial operations. The fish population were from a GlobalG.A.P. certified producer, thereby meeting the strict standards for animal welfare. An initial assessment of the clinical presentations of gross gill pathologies was carried out by scoring all eight gill arches and photographic images were taken of a range of pathologies (Figure 1). AGD score of visible AGD-related lesions was assessed based on Taylor, Muller, Cook, Kube and Elliott [6]. However, it should be noted that instead of a single score across all 16 gill hemibranchs, scores were assigned to all gill arches and a mean was taken.

Gill samples were taken for histological examination to identify lesions on the epithelium of the lamella. The gill samples were collected in methacarn (methanol-carnoy’s; 60% (*v/v*) dry methanol, 30% (*v/v*) chloroform, 10% (*v/v*) glacial acetic acid) and were processed manually for histology (washed twice in 100% methanol (30 min), twice in 100% ethanol (20 min) then cleared with two washes in xylene (15 min), impregnated with paraffin wax and sagitally and transversally sectioned at 5 µm. Sections were either stained with haematoxylin and eosin (H&E) or stained using a combined Alcian blue (pH 2.5) and periodic acid–Schiff (PAS) technique, according to Chalmers, et al. [13], i.e., the sections were de-waxed, rehydrated and immersed in Alcian blue solution (pH 2.5) for 5 min. The residual stain was then removed by washing in water and sections were oxidized in 1% (aq) periodic acid (5 min), washed (5 min) and immersed in Schiff’s reagent (20 min). Finally, all sections were washed in running tap water (10 min) and counterstained with haematoxylin Z (2 min) before being washed, dehydrated, cleared and mounted. Assessment of the digitalised histology slides was conducted blindly and independently by two histopathologists to gather descriptive information of the lesions (i.e., degenerative, inflammatory, circulatory or adaptive lesion). More specifically, the assessment was conducted using scanned histology images and following the scoring system developed by Benedicenti et al., 2019. First, all sections were quality checked for the representativeness of the cut, and only sagittal sections were included in the detail histology assessment. If the sections were damaged, autolysed or contained artefacts, they were removed from the assessment. The descriptive detail of the lesions representing degeneration, inflammation, circulatory disturbances and adaptive lesions were recorded and assessed to give a final score as in Benedicenti et al., 2019. Once the blind score was completed by the two assessors, the results were evaluated and discussed if there were any discrepancies before confirming the final histology score.

A sample from an affected area of gill (c. five filaments) was preserved in RNAlater and DNA was extracted using a Qiagen DNeasy tissue DNA extraction kit (Qiagen, Manchester, UK) following the manufacturers protocol. Taqman QPCR analysis was performed targeting the following gill pathogens: *Neoparamoeba* spp. [14], *N. perurans* [14], *Candidatus* Piscichlamydia salmonis [15], Atlantic salmon paramyxovirus (ASPV) [15], *Candidatus* Branchiomonas cysticola [16], salmon gill pox virus SGPV [17], *Desmozoon lepeophtherii* (syn. *Paranucleospora theridion*) [18], and *Ichthyobodo* spp. [19]. Ct values were normalised against a Taqman assay targeting salmon elongation factor-α [14]. In addition, Eva Green QPCR assays targeting *Tenacibaculum maritimum* [20] and total bacterial load [15] were performed.

## 3. Results

**Figure 2:**
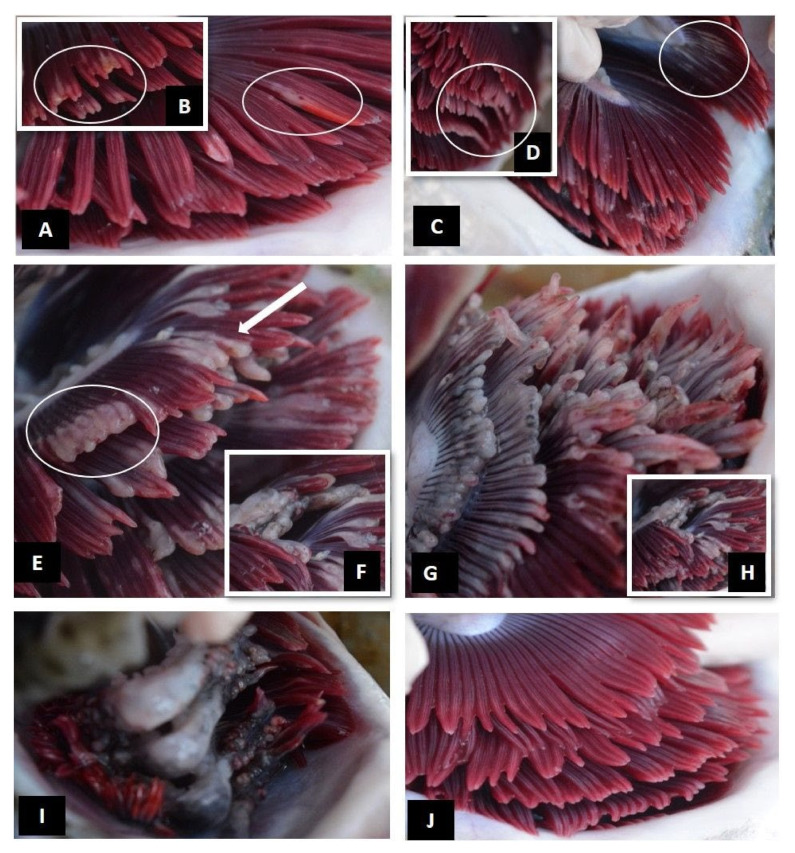
Total gill scoring system for gills of Atlantic salmon displaying multifactorial gill disease. (**A**,**B**) Score 1—very light: discrete focal white streaks or patches (**A**: white circle) on individual filaments and slight erosion/damage to distal ends of filaments (**B**: white circle); (**C**,**D**) score 2—light: more extensive coalescing white streaks or white focal patches on filaments (**C**: white circle), extended erosion/damage to distal ends of filaments (**D**: white circle); (**E**,**F**) score 3—moderate: extensive multifilamental peripheral erosion (**D**: white circle), grossly swollen or thickened filaments (**D**: arrow) with occasional areas of localised areas of necrotic tissue (**F**: white circle); (**G**,**H**) score 4—advanced: extensive, grossly swollen or thickened filaments, shortened filaments (>50% of filament length affected) with areas of necrotic tissue (**H**); (**I**) score 5—severe: widespread necrotic patches, extensive melanisation, almost total destruction of gill architecture due to extensive loss of tissue; (**J**) score 0: clear healthy gill.

The assessment of gross gill pathology allowed the development of a total gross gill scoring system, which categorized gross pathological changes on a scale of 0 (healthy) to 5 (severe) (Table 1), i.e., score 0: clear, healthy gills (Figure 2J); score 1: very light, discrete focal white streaks or patches on individual filaments and slight erosion/damage to distal ends of filaments (Figure 2A,B); score 2: more extensive coalescing white streaks or white focal patches on filaments and extended erosion/damage to distal ends of filaments (Figure 2C,D); score 3: extensive multifilamental peripheral erosion and grossly swollen or thickened filaments, with occasional areas of necrotic epithelium (Figure 2E,F); score 4: advanced: extensive, grossly shortened, swollen or thickened filaments (>50% of filament length affected) with areas of necrotic epithelium (Figure 2G,H); score 5: severe: widespread necrotic patches, extensive melanisation and almost total destruction of gill architecture due to extensive loss of tissue (Figure 2I). 

**Figure 3:**
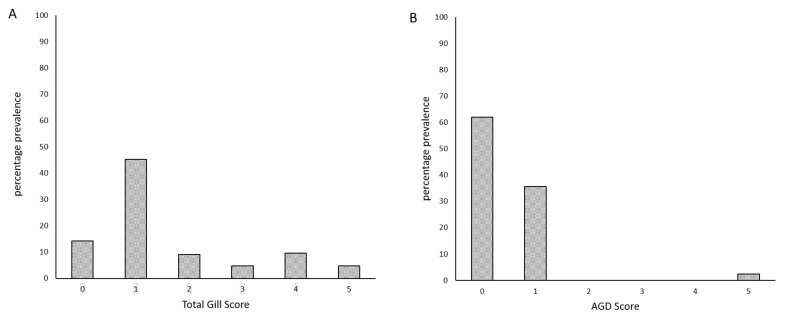
Percentage prevalence of (**A**) total gross gill scores and (**B**) AGD scores for lethally sampled fish (*n* = 42).

The prevalence of the total gross gill score and AGD score are shown in Figure 3. The AGD score system categorized a large number of fish in lesions score 0, hence assigning a false negative score to an existing lesion. The total gross gill score was able to identify advanced lesions (i.e., score 3, 4, and 5).

**Figure 4:**
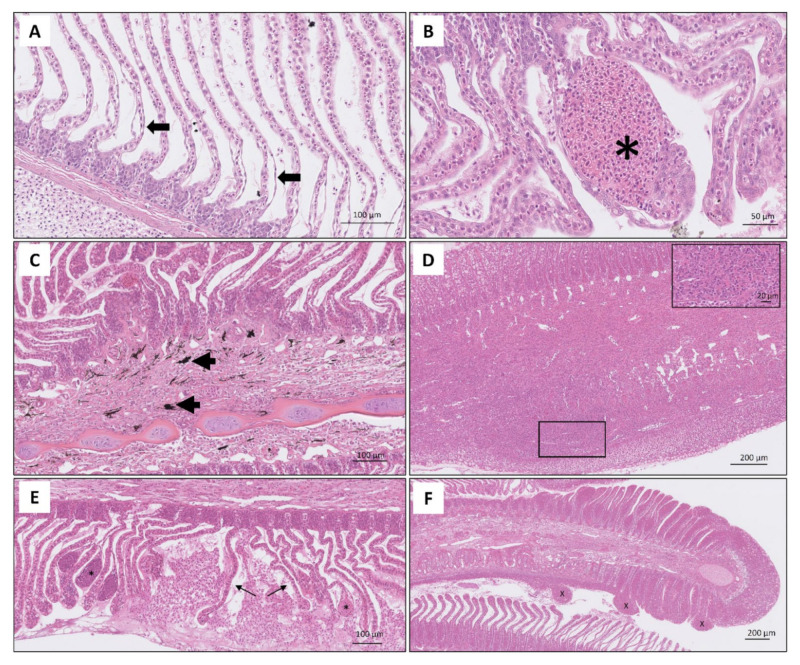
Histomicrographs of H & E-stained sections of gills showing various degrees of pathology. (**A**) Lamellae of the epithelium showing epithelial lifting; (**B**) telangiectasis resulting from rupture of pillar cells and accumulation of blood; (**C**) moderately affected gill with influx of melanin containing cells (arrowed) in the gill filament; (**D**) evidence of fusion of lamellae and formation of a diffuse granulomatous lesion along the mid length of the gill filament, Note higher magnification view of the rectangle for dense accumulation of eosinophilic granular cells and mononuclear leukocytes. No melanin containing cells were noted (**E**) degenerative lesion consists of telangiectasis (asterisk) and rupture of lamellae (arrow); (**F**) extensive hyperplastic lesions and formation of papillary lesions (x) at distal end of gill filament.

**Figure 5:**
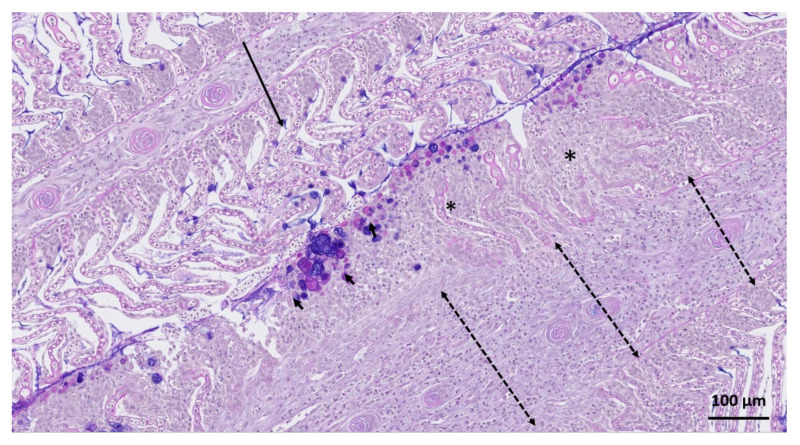
Histomicrograph of PAS-Alcian blue stained sections of gill. A mild to moderately affected gill filament (long arrow) was noted next to a filament with severe lesions (asterisk). In the severely affected filament lamellae were fused and an increased number of mucous cells were noted at the peripheral margin of the gill epithelium (solid arrows). The gill filament (dashed arrows) display a large influx of eosinophilic granular cells and mononuclear leukocytes.

The main lesions found on lamellae were chronic and varied in their degree of severity and distribution (Figure 4). Epithelial oedema, epithelial lifting (Figure 4A), sloughing, haemorrhages due to rupture of the capillaries (telangiectasias) (Figure 4B) and consolidation of blood around rupture were commonly observed in the tip and the mid shaft of the lamellae. The severe influx of eosinophilic granular cells and melanin containing cells were observed in the space between the base of the lamellae and mid shaft of the filament (Figure 4C). In a few cases, inflammatory lesions displayed chronic granulomatous changes characterized by a large influx of mononuclear cells, eosinophilic granular cells and in some instances, melanin containing cells (Figure 4D). Interlamellar fusion with excessive epithelial cell proliferation and increased number of mucous cells were clearly noted in the sections stained with PAS-Alcian blue in fish with severe pathology (Figure 5).

Of 42 samples tested, qPCR analysis for gill pathogens detected the presence of *Candidatus* Piscichlamydia salmonis [15] in 32 samples (Ct values 26.2 to 28.4), and also levels of *Ca*. Branchiomonas cysticola [16] close to the limit of detection (Ct values 33.2–35.6) in 24 of these. The same 24 samples also harboured low levels of *N. perurans*, close to the reported detection limit of 13 copies μg^−1^. In addition, high levels of *Tenacibaculum maritimum* were present in 39 samples, with Ct values <30 and Ct values 24 to 26 recorded in 7 samples. 

Detailed results including qPCR Ct values, total gross gill scores, AGD and PGD scores and histology scores, are presented in Table S1 Histology scores show a substantially higher positive correlation (0.64) with the new total gross gill score system than the traditional AGD score (0.24, AGD left and AGD right average).

## 4. Discussion and Conclusions

This novel gross gill scoring system offers a new approach to the assessment of gross pathological changes and assists in characterising the severe proliferative pathologies in gills of farmed Atlantic salmon affected by multifactorial gill disease. Gross screening is limited in its capacity to identify specific causal agents and requires further molecular and histological analyses. This scoring system can contribute to the development of robust strategies to mitigate the impacts of complex or multifactorial gill disease’ on fish health and welfare, and thereby help to prevent serious economic losses.

This case report presents a novel gross gill scoring system and its potential application in the field, hence its implications are mainly practical; the proposed scoring system provides a non-lethal, practical tool for assessing gill health of affected fish in the field. Direct comparison between total gross gill scores and AGD scores is not possible with these data due to the low prevalence of presumptive signs of AGD in the samples collected during the restricted time-period that we had access to the site; the fish were in late stages of chronic infection, and the gills were showing signs of ‘healing’ following a previous infection, as corroborated by lack of presence of *N. perurans* in the qPCR results. Nevertheless, the proposed system has potential to differentially characterize AGD lesions from complex gill disease lesions, especially in advanced stages of infection. Further studies are needed, with larger sample sizes, in order to validate and optimize gill scoring [21], and in particular for complex gill conditions of unknown aetiologies. Differences in the score weighting, as described in the Materials and Methods, may be partly responsible for the observed difference in the frequency of zero scores for the AGD and the total gross gill score, with the total gill score potentially underscoring early onset of gill lesions. This may have an impact on the future use of the novel scoring system, for example if using the total gross gill score on AGD affected populations. It should be noted that the use of individual gill arch scoring followed by taking mean values used in the current method does present practical limitations for field use due to being time consuming. The scoring system developed in the present study may be, to some extent, specific to the environment and fish sampled. This highlights the need for further validation and benchmarking of the scoring system in future gill disease outbreaks in different environments and populations.

This tool provides an easily measurable phenotype that can potentially be used as an indicator trait for resistance to multifactorial gill disease for incorporation into breeding goals. Selective breeding, using phenotypic data and either pedigree relationships or genome-wide genotypes, is an appealing complementary approach for infectious disease control and has been successfully applied in aquaculture species to improve resistance to major viral, bacterial and ectoparasitic diseases [12]. Selective breeding requires the existence of genetic variation in the trait of interest, hence, the next step is to investigate the genetic architecture of the trait defined via the phenotype of the total gross gill score, and assess its genetic correlation with the other measures of gill status, in different environments. Further, the total gross gill score is a composite phenotype describing complex gill pathology deriving from multiple causative agents. Hence, in order to develop effective epidemiological, management and genetic solutions, further studies, with larger sample sizes, are needed to address the specific aetiologies separately and explore how they correlate. Future genetic studies will investigate the genetic responses to co-infections, and explore the genetic correlations between the components of this phenotype, in order to guide its implementation in selective breeding.

It is a combination of management, conventional treatments, and genetic solutions that are needed for effective disease control, and the proposed total gross gill score can aid towards achieving these goals as a complementary diagnostic tool for field-based assessment of gill status.

## Figures and Tables

**Table 1:** Total gill scoring system to estimate severity of multifactorial gill disease in Atlantic salmon (*Salmo salar*).

Level of Infection	Total Gill Score	Description	Mean % of Gill Surface Covered
**Clear**	0	No visible pathology, healthy red coloured gills	0
**Very light**	1	Discrete focal white streaks or patches on individual filaments and slight erosion/damage to distal ends of filaments	1–5%
**Light**	2	More extensive coalescing white streaks or white focal patches on filaments, more extended erosion/damage to distal ends of filaments	5–20%
**Moderate**	3	Extensive multifilamental peripheral erosion, grossly swollen or thickened filaments with localised areas of necrotic epithelium	20–50%
**Advanced**	4	Extensive grossly swollen or thickened filaments, shortened filaments (>50% of filament length affected), pallor and areas of melanisation	50–75%
**Severe**	5	Widespread necrotic patches, extensive melanisation, almost total destruction of gill architecture due to severe loss of epithelium	>75%

## Data Availability

Data supporting reported results can be found in Table S1.

## Supplementary Materials

The following are available online at https://www.mdpi.com/article/10.3390/microorganisms9122605/s1, Table S1: qPCR results, total gross gill scores, AGD and PGD scores, and histology scores.
